# Supplementary material for: Risk of yellow fever virus transmission in the Asia-Pacific region
Source: Nat Commun. 2020 Nov 16;11:5801. doi: 10.1038/s41467-020-19625-9 (PMC7669885; doi:10.1038/s41467-020-19625-9)

Risk of yellow fever virus transmission in the Asia-Pacific region

Lucy de Guilhem de Lataillade,[1] Marie Vazeille,[1] Thomas Obadia,[2,3] Yoann Madec,[4] Laurence Mousson,[1] Basile Kamgang,[5] Chun-Hong Chen,[6] Anna-Bella Failloux,[1,*] Pei-Shi Yen[1,*]

[1] Arboviruses and Insect Vectors Unit, Institut Pasteur, Paris, France

[2] Bioinformatics and Biostatistics Hub, Institut Pasteur, USR 3756, CNRS, Paris, France

[3] Malaria Unit: Parasites and Hosts, Institut Pasteur, Paris, France

[4] Emerging Diseases Epidemiology Unit, Institut Pasteur, Paris, France

[5] Centre for Research in Infectious Diseases, Department of Medical Entomology, Yaoundé, Cameroon

[6] National Health Research Institutes, Institute of Infectious Diseases and Vaccinology, Miaoli, Taiwan

* Co-corresponding authors

Correspondence to: Pei-Shi Yen (pei-shi.yen@pasteur.fr), Anna-Bella Failloux (anna-bella.failloux@pasteur.fr)

Supplementary Figure 1. Dissemination and transmission efficiencies of *Aedes aegypti* populations at 14 and 21 days post-infection (dpi). After an infectious blood meal at a titer of 10^7ffu/mL of YFV, mosquitoes were processed as described in Fig. 2. Head and saliva were titrated on C6/36 cells in 96-well plates to estimate dissemination (a, b) and transmission (c, d). Dissemination efficiency (DE) refers to the percentage of mosquitoes with an infected head (containing viral particles having disseminated in the general cavity after crossing successfully the midgut) among tested mosquitoes. Transmission efficiency (TE) corresponds to the percentage of mosquitoes with infectious saliva (viral particles having successively crossed the two anatomical barriers, midgut and salivary glands) among tested mosquitoes. Stars indicate statistical significance of comparisons by Fisher's exact test (two-sided test; *P ≤ 0·05, ** P ≤ 0·01, **** P ≤ 0·0001). S1a: **** P ≤ 0·0001, ** P = 0·003; S1b: **** P ≤ 0·0001, * P = 0·038; S1c: **** P ≤ 0·0001; S1d: **** P ≤ 0·0001. ns (non-significant) indicates the lack of statistical significance (P > 0·05).

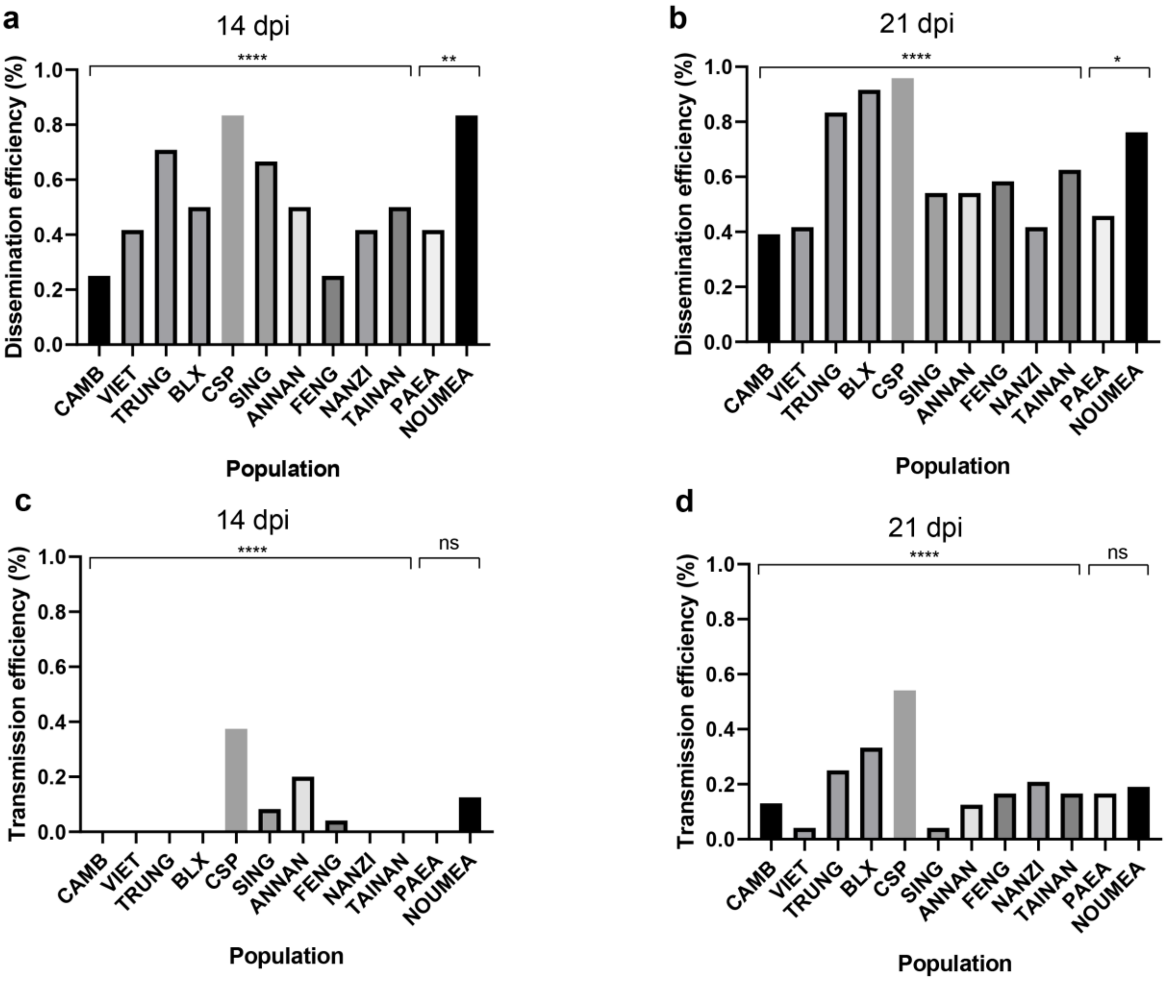

Supplementary Figure 2. Dissemination and transmission efficiencies of *Aedes albopictus* populations at 14 and 21 days post-infection (dpi). After an infectious blood meal at a titer of 10^7ffu/mL of YFV, mosquitoes were processed as described in Fig. 2. Head and saliva were titrated on C6/36 cells in 96-well plates to estimate dissemination (a, b) and transmission (c, d). Dissemination efficiency (DE) refers to the percentage of mosquitoes with an infected head (containing viral particles having disseminated in the general cavity after crossing successfully the midgut) among tested mosquitoes. Transmission efficiency (TE) corresponds to the percentage of mosquitoes with infectious saliva (viral particles having successively crossed the two anatomical barriers, midgut and salivary glands) among tested mosquitoes. Stars indicate statistical significance of comparisons by Fisher's exact test (two-sided test; *** $P \leq 0{\cdot}001$). ns (non-significant) indicates the lack of statistical significance ($P > 0{\cdot}05$).

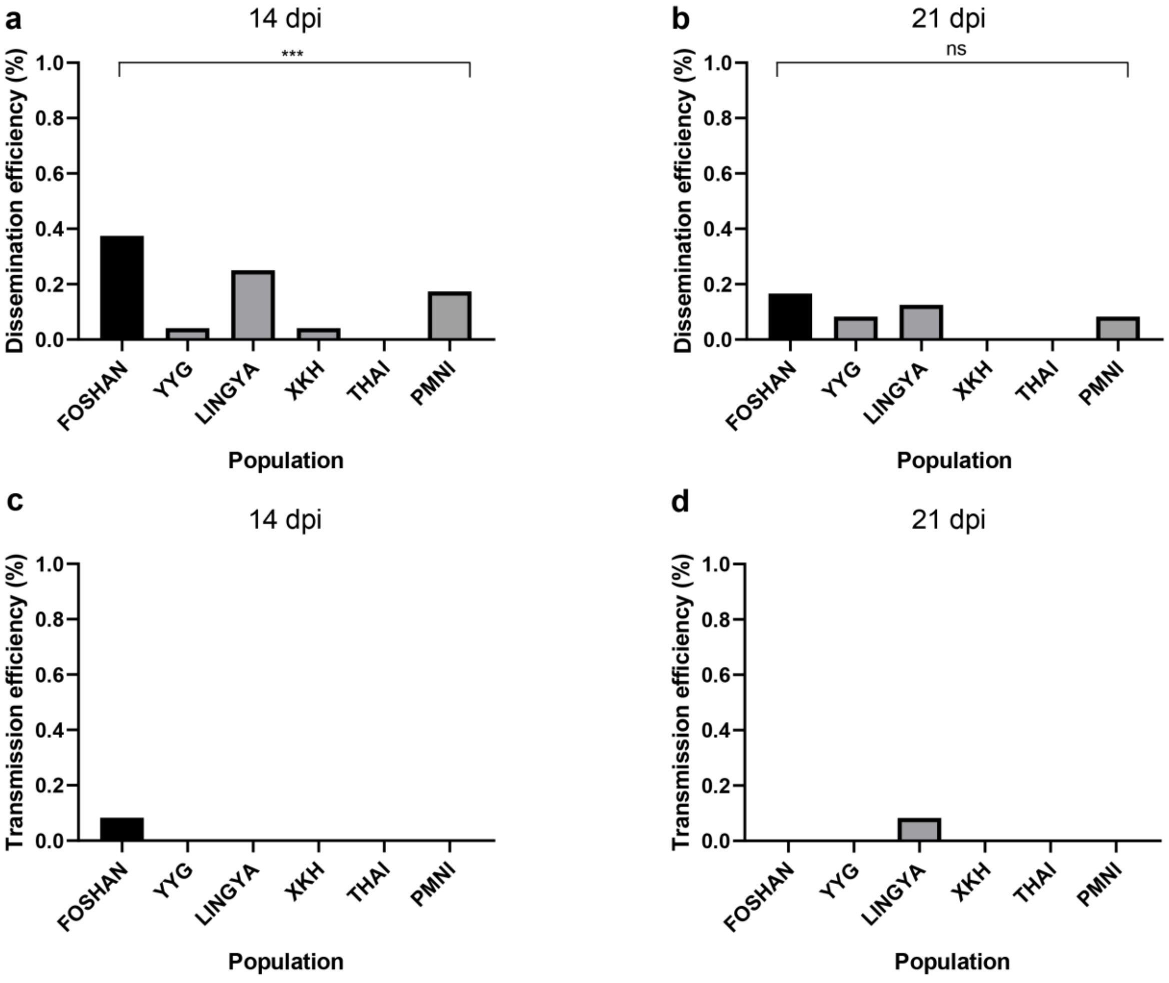

Supplementary Figure 3. Viral loads in body, head and saliva of *Aedes aegypti* from Asia (a), Africa (b), and Pacific region (c). Twenty-one days after an infectious blood meal provided at 10^7 ffu/mL, body (abdomen and thorax), head, and saliva were processed for titration on C6/36 cells in 96-well plates. Viral loads were expressed in number of viral particles per sample. 48 mosquitoes from Asia, 10 from Africa and 6 from Pacific region were analyzed. Stars indicate statistical significance of comparisons by Kruskal-Wallis test (two-sided test; ** P ≤ 0·01, *** P ≤ 0·001, **** P ≤ 0·0001*)*. S3a: **** P ≤ 0·0001; S3b: *** P ≤ 0·001: S3c: ** P = 0·0022.

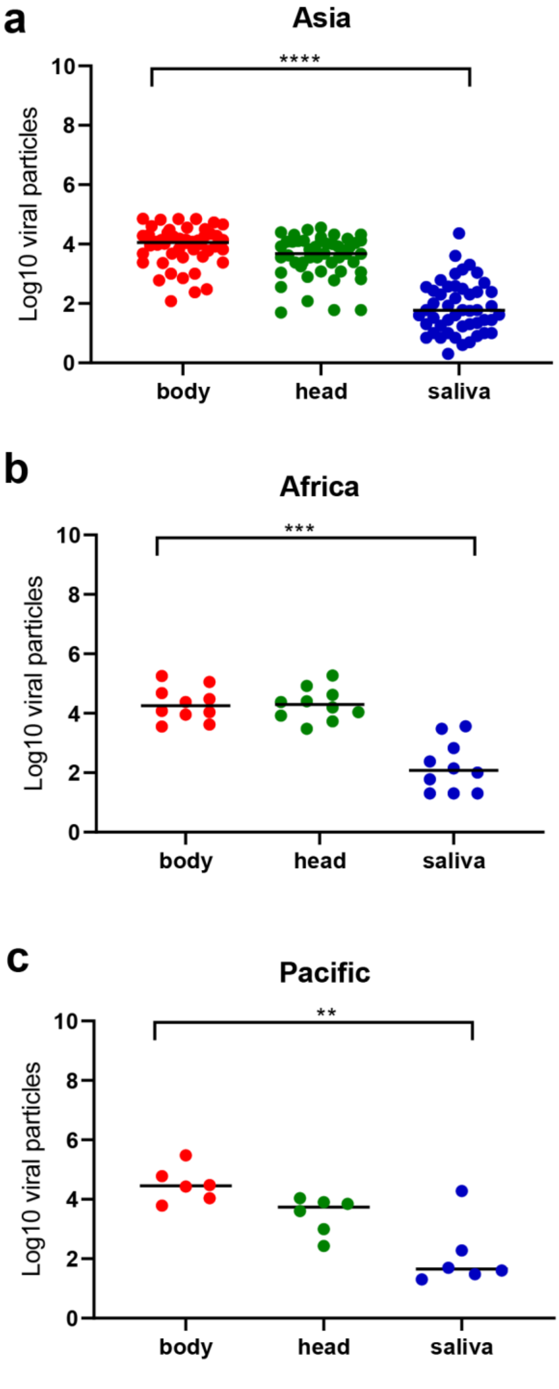

Supplementary Figure 4. Correlation between viral loads in body and head (a), body and saliva (b), and head and saliva (c). Twenty-one days after an infectious blood meal provided at 10^7 ffu/mL, body (abdomen and thorax), head, and saliva were processed for titration on C6/36 cells in 96-well plates. Viral loads were expressed in Log_{10} number of viral particles per sample. P values indicate statistical significance of associations by Pearson's correlation coefficient test (two-sided test; significant when $P < 0{\cdot}05$; non-significant when $P > 0{\cdot}05$).

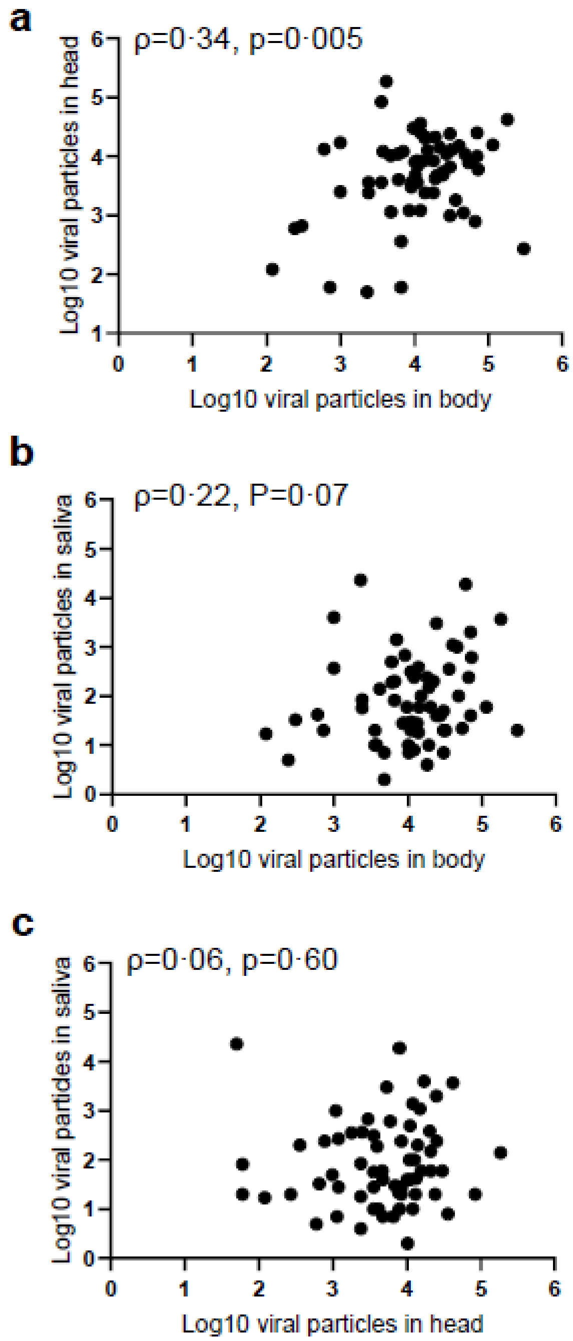

Supplement: Supplementary file 1 — Supplementary Information [file 41467_2020_19625_MOESM1_ESM.pdf]
