# Supplementary material for: Risk of yellow fever virus transmission in the Asia-Pacific region
Source: Nat Commun. 2020 Nov 16;11:5801. doi: 10.1038/s41467-020-19625-9 (PMC7669885; doi:10.1038/s41467-020-19625-9)
Supplement: Supplementary file 3 — Descriptions of Additional Supplementary Files [file 41467_2020_19625_MOESM3_ESM.pdf]

Descriptions of Additional Supplementary Files

Supplementary Data 1

Description: Infection, dissemination and transmission of YFV by mosquitoes in the Asia-Pacific region.

Supplementary Data 2

Description: Viral loads in body, head and saliva of mosquitoes capable of YFV transmission.
