# Supplementary material for: Risk of yellow fever virus transmission in the Asia-Pacific region
Source: Nat Commun. 2020 Nov 16;11:5801. doi: 10.1038/s41467-020-19625-9 (PMC7669885; doi:10.1038/s41467-020-19625-9)
Supplement: Supplementary file 6 — Reporting Summary [file 41467_2020_19625_MOESM6_ESM.pdf]

nature research

Corresponding author(s): Anna-Bella Failloux, Pei-Shi Yen

Last updated by author(s): Oct 15, 2020

# Reporting Summary

Nature Research wishes to improve the reproducibility of the work that we publish. This form provides structure for consistency and transparency in reporting. For further information on Nature Research policies, see our Editorial Policies and the Editorial Policy Checklist.

## Statistics

For all statistical analyses, confirm that the following items are present in the figure legend, table legend, main text, or Methods section.

| n/a | Confirmed | |
|---|---|---|
| ☐ | ☒ | The exact sample size ($n$) for each experimental group/condition, given as a discrete number and unit of measurement |
| ☒ | ☐ | A statement on whether measurements were taken from distinct samples or whether the same sample was measured repeatedly |
| ☐ | ☒ | The statistical test(s) used AND whether they are one- or two-sided<br>*Only common tests should be described solely by name; describe more complex techniques in the Methods section.* |
| ☒ | ☐ | A description of all covariates tested |
| ☒ | ☐ | A description of any assumptions or corrections, such as tests of normality and adjustment for multiple comparisons |
| ☐ | ☒ | A full description of the statistical parameters including central tendency (e.g. means) or other basic estimates (e.g. regression coefficient) AND variation (e.g. standard deviation) or associated estimates of uncertainty (e.g. confidence intervals) |
| ☐ | ☒ | For null hypothesis testing, the test statistic (e.g. $F$, $t$, $r$) with confidence intervals, effect sizes, degrees of freedom and $P$ value noted<br>*Give P values as exact values whenever suitable.* |
| ☒ | ☐ | For Bayesian analysis, information on the choice of priors and Markov chain Monte Carlo settings |
| ☒ | ☐ | For hierarchical and complex designs, identification of the appropriate level for tests and full reporting of outcomes |
| ☒ | ☐ | Estimates of effect sizes (e.g. Cohen's $d$, Pearson's $r$), indicating how they were calculated |

*Our web collection on statistics for biologists contains articles on many of the points above.*

## Software and code

Policy information about availability of computer code

| | |
|---|---|
| Data collection | No software was used. |
| Data analysis | Statistical analyses were performed using the Stata software (version 10.0, StataCorp LP, Texas, USA). |

For manuscripts utilizing custom algorithms or software that are central to the research but not yet described in published literature, software must be made available to editors and reviewers. We strongly encourage code deposition in a community repository (e.g. GitHub). See the Nature Research guidelines for submitting code & software for further information.

## Data

Policy information about availability of data

All manuscripts must include a data availability statement. This statement should provide the following information, where applicable:

- Accession codes, unique identifiers, or web links for publicly available datasets
- A list of figures that have associated raw data
- A description of any restrictions on data availability

The data that support the findings of this study are available as supplementary information files (Supplementary Data1, Supplementary Data 2)

# Field-specific reporting

# Life sciences study design

All studies must disclose on these points even when the disclosure is negative.

| | |
|---|---|
| Sample size | The sample size is determined based on our experience in vector competence studies to obtain statistical significance and reproductibility; we examined 20-24 mosquitoes at each time point for each population. |
| Data exclusions | No data were excluded from the analysis. |
| Replication | We used mainly field-collected mosquitoes which usually feed with difficulty in BSL3 conditions. The number of fed mosquitoes is usually low allowing us to run only one single experiment with 20-24 mosquitoes examined at each time point. |
| Randomization | Mosquitoes of the same cage were sorted randomly to constitute boxes of 60 female adults for infection.<br>After infection, fully fed mosquitoes were maintained in cardboard boxes until examination. 20-24 surviving mosquitoes were randomly chosen to be examined at 14 and 21 days post-infection. All these statements are in the ms. |
| Blinding | Blinding is not applicable to our study; all mosquitoes are exposed to an infectious blood meal. |

# Reporting for specific materials, systems and methods

We require information from authors about some types of materials, experimental systems and methods used in many studies. Here, indicate whether each material, system or method listed is relevant to your study. If you are not sure if a list item applies to your research, read the appropriate section before selecting a response.

## Materials & experimental systems

| n/a | Involved in the study | |
|---|---|---|
| ☐ | ☒ | Antibodies |
| ☒ | ☐ | Eukaryotic cell lines |
| ☒ | ☐ | Palaeontology and archaeology |
| ☐ | ☒ | Animals and other organisms |
| ☒ | ☐ | Human research participants |
| ☒ | ☐ | Clinical data |
| ☒ | ☐ | Dual use research of concern |

## Methods

| n/a | Involved in the study | |
|---|---|---|
| ☒ | ☐ | ChIP-seq |
| ☒ | ☐ | Flow cytometry |
| ☒ | ☐ | MRI-based neuroimaging |

## Antibodies

| | |
|---|---|
| Antibodies used | - YFV specific primary antibody (NB100-64510, Novusbio, CO, USA)<br>- a fluorescent-conjugated secondary antibody (A-11029, Life Technologies, CA, USA) |
| Validation | The YFV primary antibody is a commercial product; its validation as YFV-ab can be found on the supplier's website (https://www.novusbio.com/products/yellow-fever-virus-antibody-Og5_nb100-64510#PublicationSection). A publication is attached. |

## Animals and other organisms

Policy information about studies involving animals; ARRIVE guidelines recommended for reporting animal research

| | |
|---|---|
| Laboratory animals | We used:<br>- mice (mus musculus), Charles River, IOPS female OF1 (20/22g), aged between 6 weeks and 2 months and maintained in an animal facility under standard conditions (23°C and 14:10 light/dark cycle) at Institut Pasteur, to feed mosquitoes for obtaining eggs<br>- rabbit (Oryctolagus cuniculus), Charles River, New Zealand White male (2.7 - 3 kg), aged between 3 months and 2 years and maintained in an animal facility under standard conditions (23°C and 14:10 light/dark cycle) at Institut Pasteur, for blood to run artificial infections of mosquitoes. |
| Wild animals | The study did not involve wild animals. |
| Field-collected samples | Mosquito eggs were collected using ovitraps placed in each site. After collection, eggs were were immersed in water for hatching. Larvae were reared in pans containing one liter of water and fed with yeast tablets. Adults were maintained in controlled conditions (28°C, 70% relative humidity, 16:8 hour (Light:Dark) photoperiod) until infection. |
| Ethics oversight | Work on animals was performed in compliance with French and European regulations on care and protection of laboratory animals (EC Directive 2010/63, French Law 2013-118, February 6th, 2013). All experiments were approved by the Ethics Committee #89 and registered under the reference APAFIS (Autorisation de Projet utilisant des Animaux à des FIns Scientifiques) #6573-201606l412077987 v2. |

Note that full information on the approval of the study protocol must also be provided in the manuscript.
